# Supplementary material for: The meiotic TERB1-TERB2-MAJIN complex tethers telomeres to the nuclear envelope
Source: Nat Commun. 2019 Feb 4;10:564. doi: 10.1038/s41467-019-08437-1 (PMC6361898; doi:10.1038/s41467-019-08437-1)
Supplement: Supplementary file 1 — Supplementary Information [file 41467_2019_8437_MOESM1_ESM.pdf]

Supplementary Information

The meiotic TERB1-TERB2-MAJIN complex tethers telomeres to the nuclear envelope

Y. Wang, Y. Chen et al.

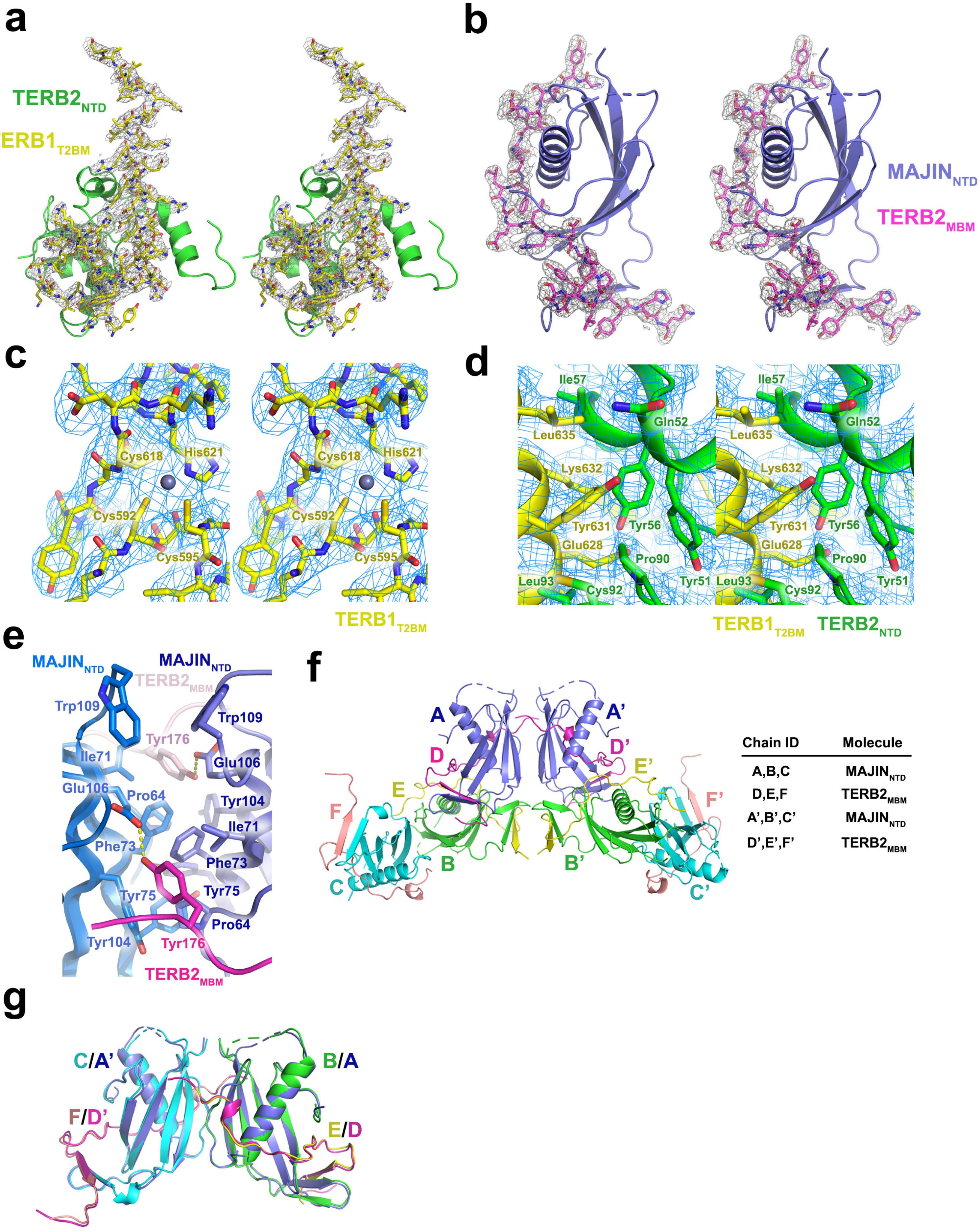
a
TERB2$_{NTD}$
TERB1$_{T2BM}$
b
MAJIN$_{NTD}$
TERB2$_{MBM}$
c
Cys618
His621
Cys592
Cys595
TERB1$_{T2BM}$
d
Ile57
Gln52
Leu635
Lys632
Tyr631
Tyr56
Glu628
Pro90
Leu93
Cys92
Tyr51
TERB1$_{T2BM}$
TERB2$_{NTD}$
e
MAJIN$_{NTD}$
MAJIN$_{NTD}$
TERB2$_{MBM}$
Trp109
Trp109
Tyr176
Ile71
Glu106
Glu106
Pro64
Tyr104
Ile71
Phe73
Phe73
Tyr75
Tyr75
Pro64
Tyr104
Tyr176
TERB2$_{MBM}$
f
A
A'
D
D'
E
E'
F
F'
B
B'
C
C'
Chain ID
Molecule
A,B,C
MAJIN$_{NTD}$
D,E,F
TERB2$_{MBM}$
A',B',C'
MAJIN$_{NTD}$
D',E',F'
TERB2$_{MBM}$
g
C/A'
B/A
F/D'
E/D

Supplementary Figure 1

Stereo views of the TERB1$_{T2BM}$-TERB2$_{NTD}$ complex and the TERB2$_{MBM}$-MAJIN$_{NTD}$ complex

(a) Stereo view of the TERB1$_{T2BM}$-TERB2$_{NTD}$ complex. TERB1$_{T2BM}$ is shown as a stick model (yellow) with the electron density map (1.0 σ) and TERB2$_{NTD}$ is shown as green ribbon. (**b**) Stereo view of the TERB2$_{MBM}$-MAJIN$_{NTD}$ complex. TERB2$_{MBM}$ is shown as a stick model (magenta) with the electron density map (1.0 σ) and MAJIN$_{NTD}$ is shown as blue ribbon. **(c)** Stereo view of the density map (1.0 σ) of the zinc-finger region in TERB1$_{T2BM}$. **(d)** Stereo view of the density map (1.0 σ) of the interface between TERB2$_{NTD}$ (green) and TERB1$_{T2BM}$ (yellow). **(e)** Detailed interactions show a hydrophobic interface between the two TERB2$_{MBM}$-MAJIN$_{NTD}$ protomers. **(f)** Ribbon diagram of six TERB2$_{MBM}$-MAJIN$_{NTD}$ protomers from two adjacent asymmetric units. In one asymmetric unit, two TERB2$_{MBM}$-MAJIN$_{NTD}$ protomers (chain B-E and chain C-F) form a heterotetramer, and the third protomer (chain A-D) forms another identical heterotetramer with its crystallograhically symmetric counterpart (chain A'-D'). **(g)** Superimposition of the two heterotetramers in (f).

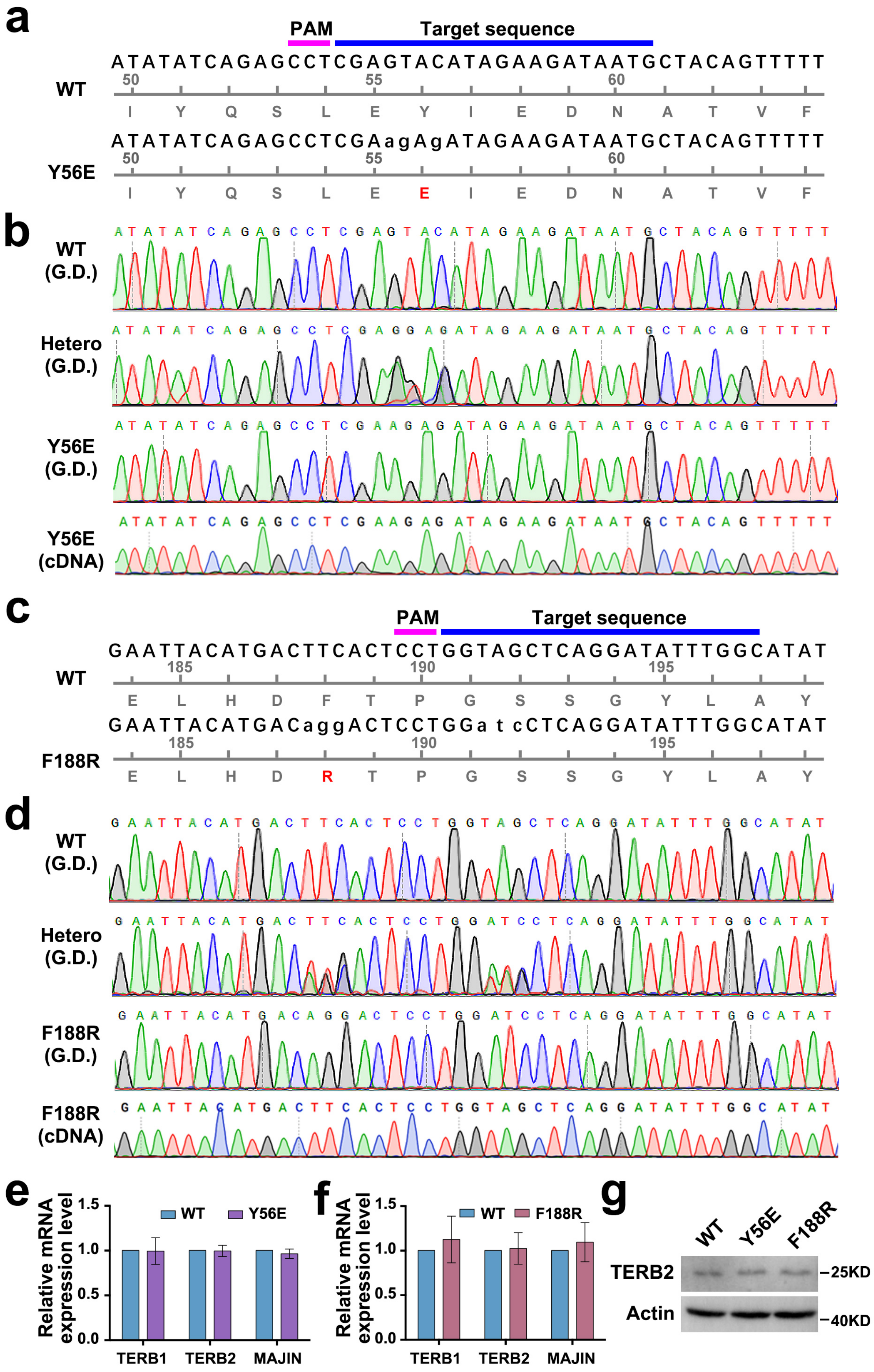

a
PAM
Target sequence
ATATATCAGAGCCTCGAGTACATAGAAGATAATGCTACAGTTTTT
WT
50
55
60
I Y Q S L E Y I E D N A T V F
ATATATCAGAGCCTCGAagAgATAGAAGATAATGCTACAGTTTTT
Y56E
I Y Q S L E E I E D N A T V F
b
WT (G.D.)
Hetero (G.D.)
Y56E (G.D.)
Y56E (cDNA)
c
PAM
Target sequence
GAATTACATGACTTCACTCCTGGTAGCTCAGGATATTTGGCATAT
WT
185
190
195
E L H D F T P G S S G Y L A Y
GAATTACATGACaggACTCCTGGa t cCTCAGGATATTTGGCATAT
F188R
E L H D R T P G S S G Y L A Y
d
WT (G.D.)
Hetero (G.D.)
F188R (G.D.)
F188R (cDNA)
e
Relative mRNA expression level
WT
Y56E
TERB1
TERB2
MAJIN
f
Relative mRNA expression level
WT
F188R
TERB1
TERB2
MAJIN
g
WT
Y56E
F188R
TERB2
Actin
–25KD
–40KD

Supplementary Figure 2

Targeted disruption of the TTM complex in mice using the CRISPR/Cas9 method

(a) The Cas9/sgRNA-targeting site for mouse *Terb2*^{Y56E}. The sgRNA-targeting site and the protospacer adjacent motif (PAM) are indicated above the sequence. The desired mutations on genomic DNA are indicated by lower-case letters. The altered amino acid residue is indicated in red color. **(b)** *Terb2*^{Y56E} mouse offspring were genotyped by tail biopsy with PCR and DNA sequencing of the mutation loci. Testis cDNA library from homozygous mice was also subjected to PCR and DNA sequencing to confirmed the desired mutation. **(c)** The Cas9/sgRNA-targeting site for mouse *Terb2*^{F188R}. The sgRNA-targeting site and the PAM are indicated above the sequence. The desired mutations on genomic DNA are indicated by lower-case letters, and the altered amino acid residue is indicated in red color. **(d)** *Terb2*^{F188R} mouse offspring were genotyped by tail biopsy with PCR and DNA sequencing of the mutation loci. Testis cDNA library from homozygous mice was also subjected to PCR and DNA sequencing to confirmed the desired mutation. **(e** and **f)** Relative mRNA levels of *Terb1*, *Terb2* and *Majin* were determined by RT-qPCR with testes from 2-week-old WT and *Terb2*^{Y56E} (e) or *Terb2*^{F188R} (f) littermates using GAPDH as the loading control. The averaged value for WT was normalized to 1. Results were from three independent experiments. Error bars: standard deviation; n=3. **(g)** Western blot of TERB2 in testes of 2-week-old mice. β-actin was used as the loading control. Source data are provided as a Source Data file.

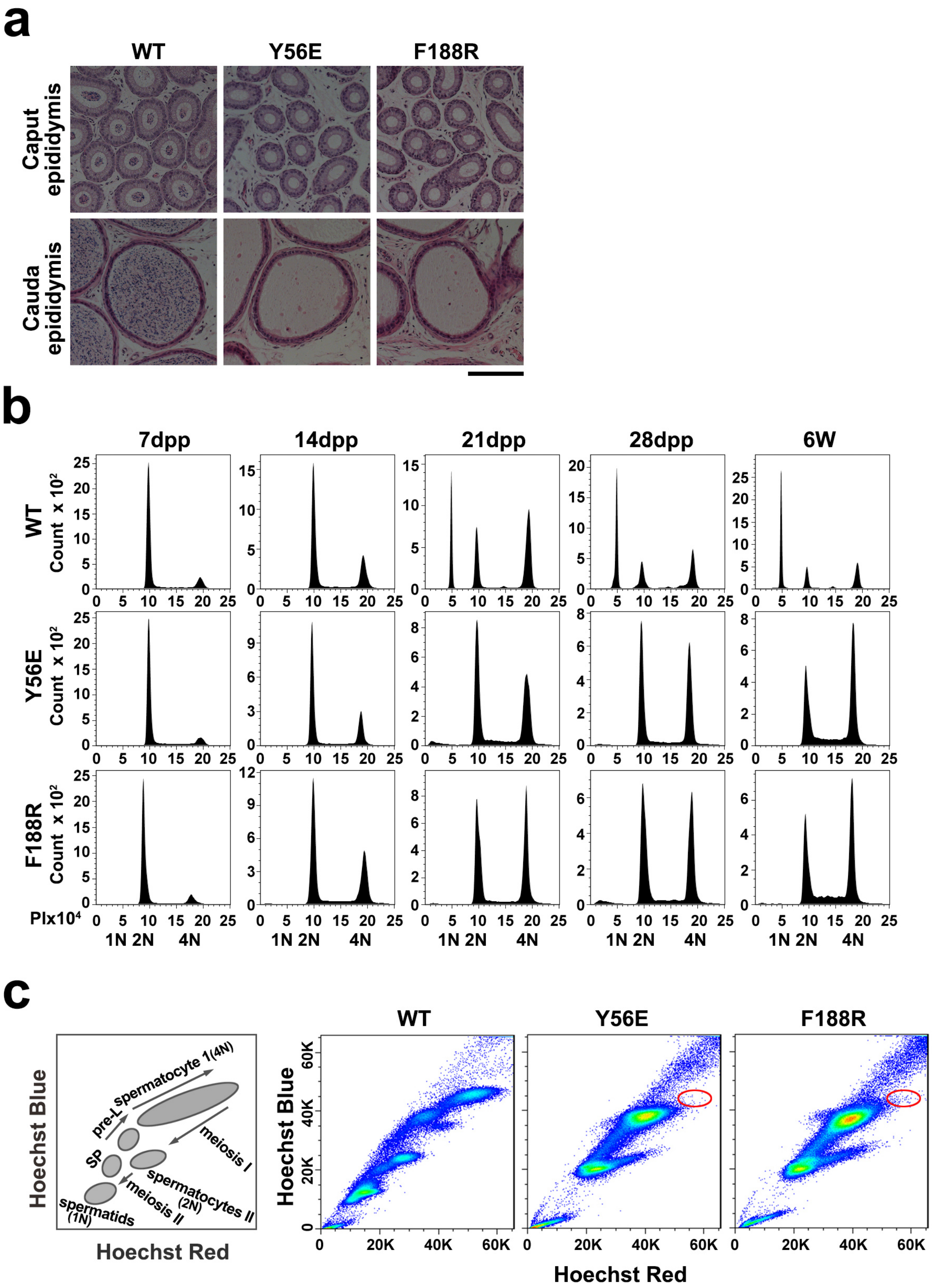
a
WT
Y56E
F188R
Caput epididymis
Cauda epididymis
b
7dpp
14dpp
21dpp
28dpp
6W
WT Count x 10²
Y56E Count x 10²
F188R Count x 10²
PIx10⁴
1N 2N 4N
c
Hoechst Blue
Hoechst Red
Spermatocyte 1(4N)
pre-L
SP
meiosis I
spermatocytes II (2N)
meiosis II
spermatids (1N)
WT
Y56E
F188R
0
20K
40K
60K

Supplementary Figure 3

Disruption of the TERB1-TERB2 and the TERB2-MAJIN interactions causes a meiotic arrest at Pachytene-like stage

(a) Hematoxylin and eosin-stained histological cross-sections of epididymis from 6-week-old male mice. There is no sperm in the epididymis from the *Terb2*^{Y56E} and *Terb2*^{F188R} mice. Scale bars, 100 μm. **(b)** Propidium iodide (PI) fluorescence flow analysis of fixed testicular cells from WT, *Terb2*^{Y56E}, and *Terb2*^{F188R} mice at different ages. Cells were stained by propidium iodide (PI). 1N, haploid; 2N, diploid; 4N, tetraploid. **(c)** Hoechst 33342 fluorescence flow analysis of testicular cells from 6-week-old mice. The lack of more advanced tetraploid spermatocytes in the mutant mice is indicated in the regions circled in red. SP: Spermatogonia, pre-L: pre-Leptotene.

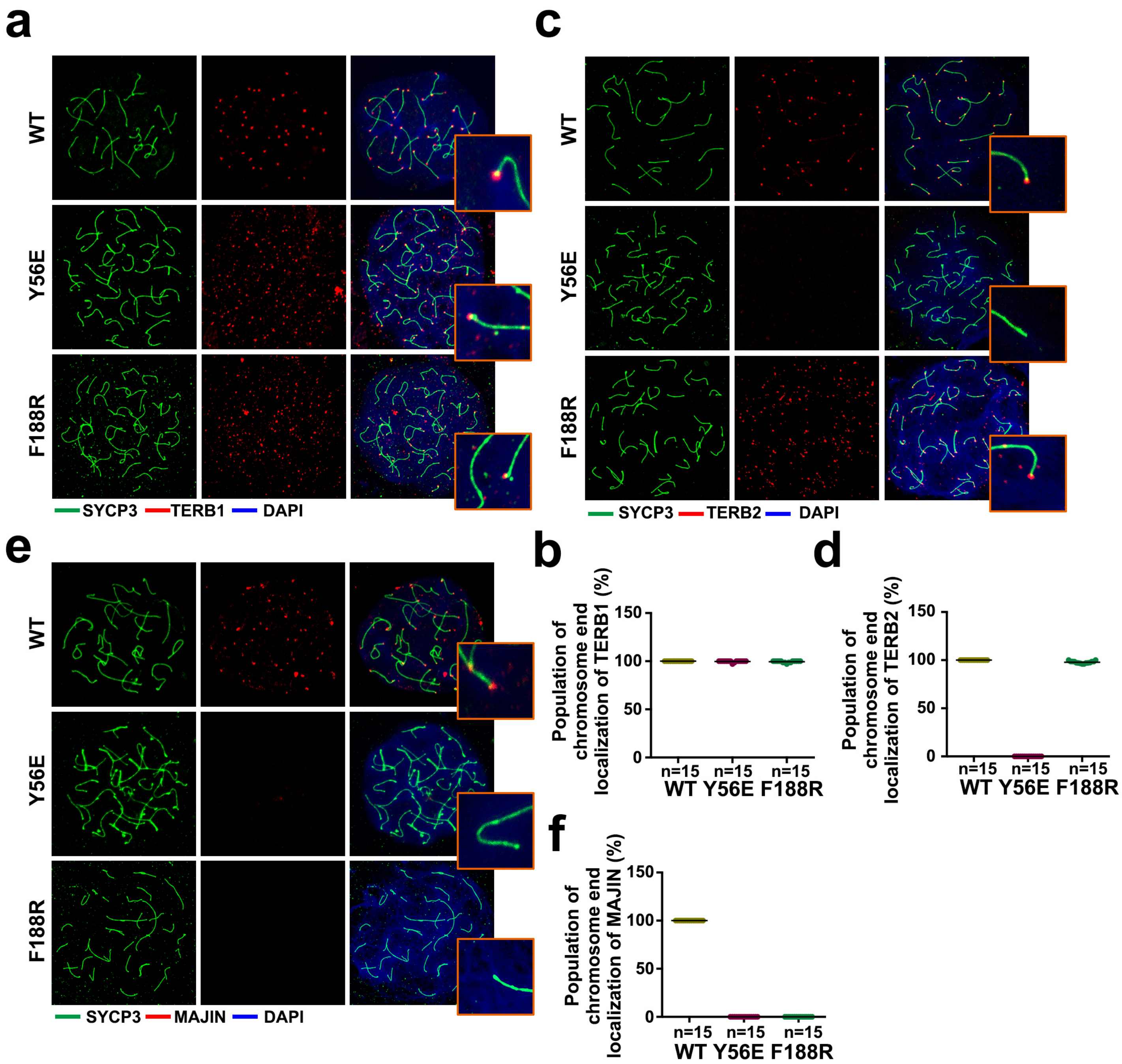
a
c
e
b
d
f
WT
Y56E
F188R
SYCP3
TERB1
DAPI
SYCP3
TERB2
DAPI
SYCP3
MAJIN
DAPI
Population of chromosome end localization of TERB1 (%)
Population of chromosome end localization of TERB2 (%)
Population of chromosome end localization of MAJIN (%)
150
100
50
0
n=15
n=15
n=15
WT Y56E F188R

Supplementary Figure 4

Telomeric localization of TERB1, TERB2 and MAJIN in spermatocytes

(a, c, e) Representative images showing the telomeric distribution of TERB1, TERB2 and MAJIN in WT pachytene spermatocytes or mutant spermatocytes at the most advanced stage. Spermatocyte spreads from 6-week-old mice of the indicated genotypes were immunostained for SYCP3 as well as TERB1 (a), or TERB2 (c) or MAJIN (e). DNA was stained by DAPI. The zoom-in views show one chromosome end from each spread. **(b, d, f)** Dot plot showing population of chromosome ends with TERB1 foci (b), TERB2 foci (d), or MAJIN foci (e) in one nucleus. n=15 nuclei were analyzed for each indicated genotypes. Source data are provided as a Source Data file.

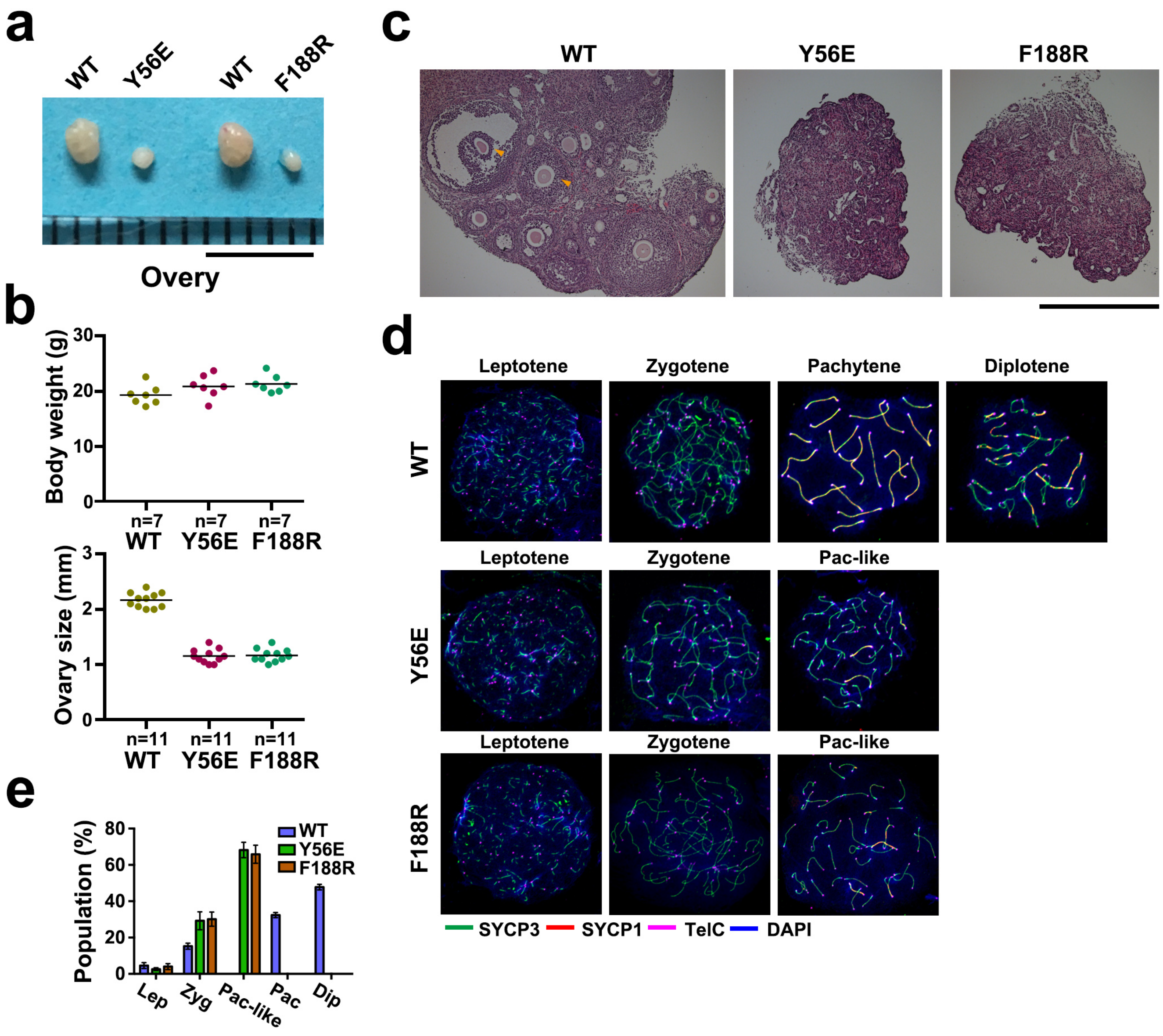
a
WT
Y56E
WT
F188R
Overy
b
Body weight (g)
30
20
10
0
n=7
n=7
n=7
WT
Y56E
F188R
Ovary size (mm)
3
2
1
0
n=11
n=11
n=11
WT
Y56E
F188R
c
WT
Y56E
F188R
d
Leptotene
Zygotene
Pachytene
Diplotene
WT
Leptotene
Zygotene
Pac-like
Y56E
Leptotene
Zygotene
Pac-like
F188R
SYCP3
SYCP1
TelC
DAPI
e
Population (%)
80
60
40
20
0
WT
Y56E
F188R
Lep
Zyg
Pac-like
Pac
Dip

Supplementary Figure 5

Oocytogenesis is disrupted in TERB2-deficient mice

(a) Ovaries from 6-week-old mice of the indicated genotypes. Scale bars, 5 mm. **(b)** Body weight (top) and ovary size (the longest diameter) (bottom) of 6-week-old female mice of the indicated genotypes. **(c)** Ovary tissue sections from adult female mice stained with hematoxylin and eosin. Arrowheads indicate oocytes and growing follicles that are not observed in mutant ovaries. Scale bars, 0.5 mm. **(d)** Representative fetal oocytes from 19-dpc embryos at different stages stained with DAPI and antibodies of SYCP3 and SYCP1. Pac-like: Pachytene-like. **(e)** Quantification of the frequencies of meiotic stages shown in (d). Data are from three independent experiments of different mice. Error bars: standard deviation; n=3. More than 60 nuclei from each mouse were counted. Lep: Leptotene, Zyg: Zygotene, Pac-like: Pachytene-like, Pac: Pachytene, Dip: Diplotene, Dia: Diakinesis. Source data are provided as a Source Data file.

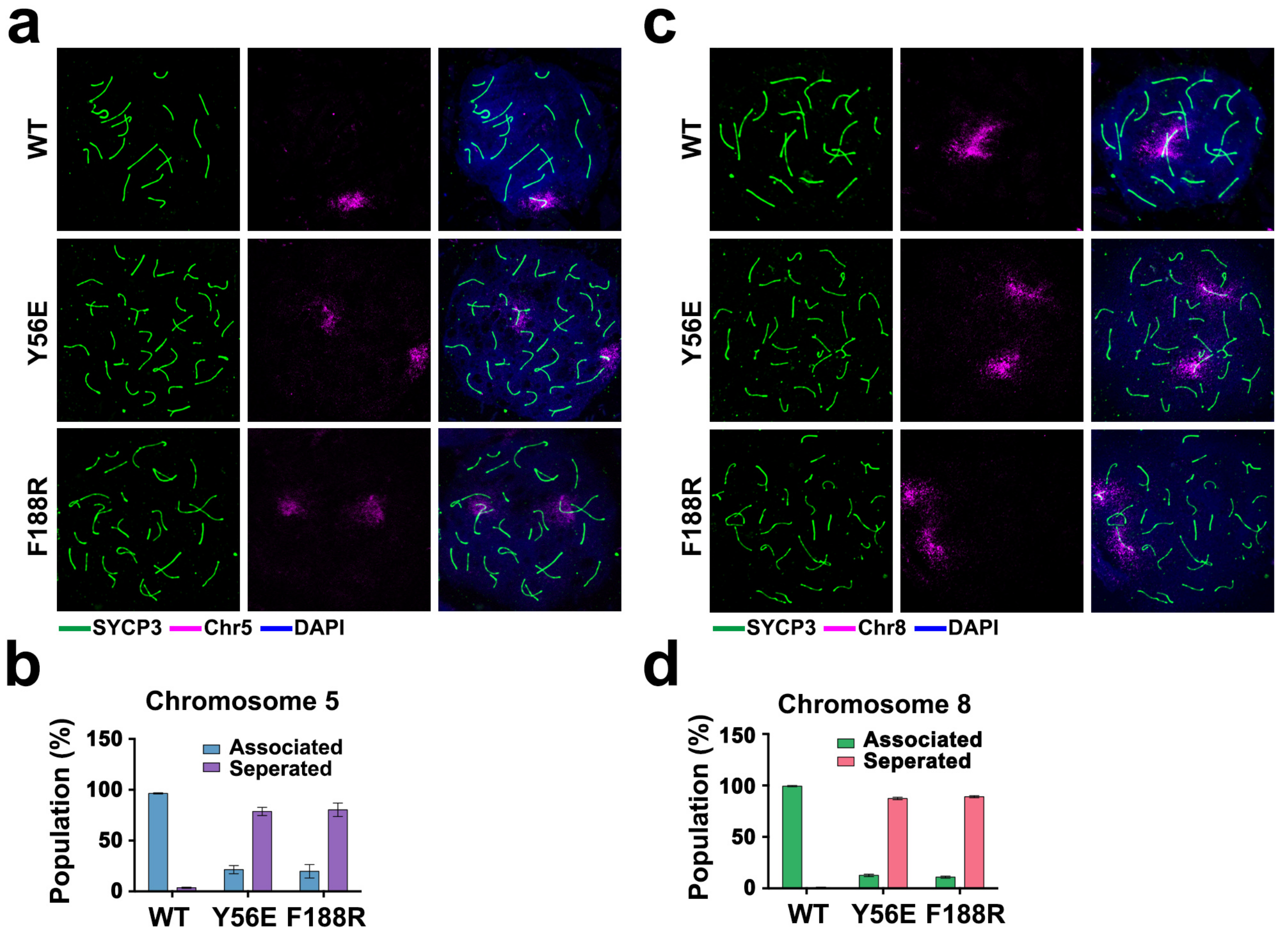

a
WT
Y56E
F188R
SYCP3
Chr5
DAPI
b
Chromosome 5
Population (%)
150
100
50
0
Associated
Seperated
WT
Y56E
F188R
c
WT
Y56E
F188R
SYCP3
Chr8
DAPI
d
Chromosome 8
Population (%)
150
100
50
0
Associated
Seperated
WT
Y56E
F188R

Supplementary Figure 6

Disruption of the TERB1-TERB2 and the TERB2-MAJIN interactions leads to impairment of homologous association

(a) Immunostaining of SYCP3 and FISH detection of chromosome 5 on spermatocyte spreads from adult testes of different genotypes. **(b)** Quantification of the population of associated or separated chromosome 5. Data are from three independent experiments with different mice. Error bars: standard deviation; n=3. For each mouse, more than 50 spreads were counted for quantification. **(c)** IF-FISH analysis of chromosome 8 and SYCP3 on adult spermatocyte spreads. **(d)** Quantification of the population of associated or separated chromosome 8. Data are from three independent experiments with different mice. Error bars: standard deviation; n=3. For each mouse, more than 50 spreads were counted for quantification. Source data are provided as a Source Data file.

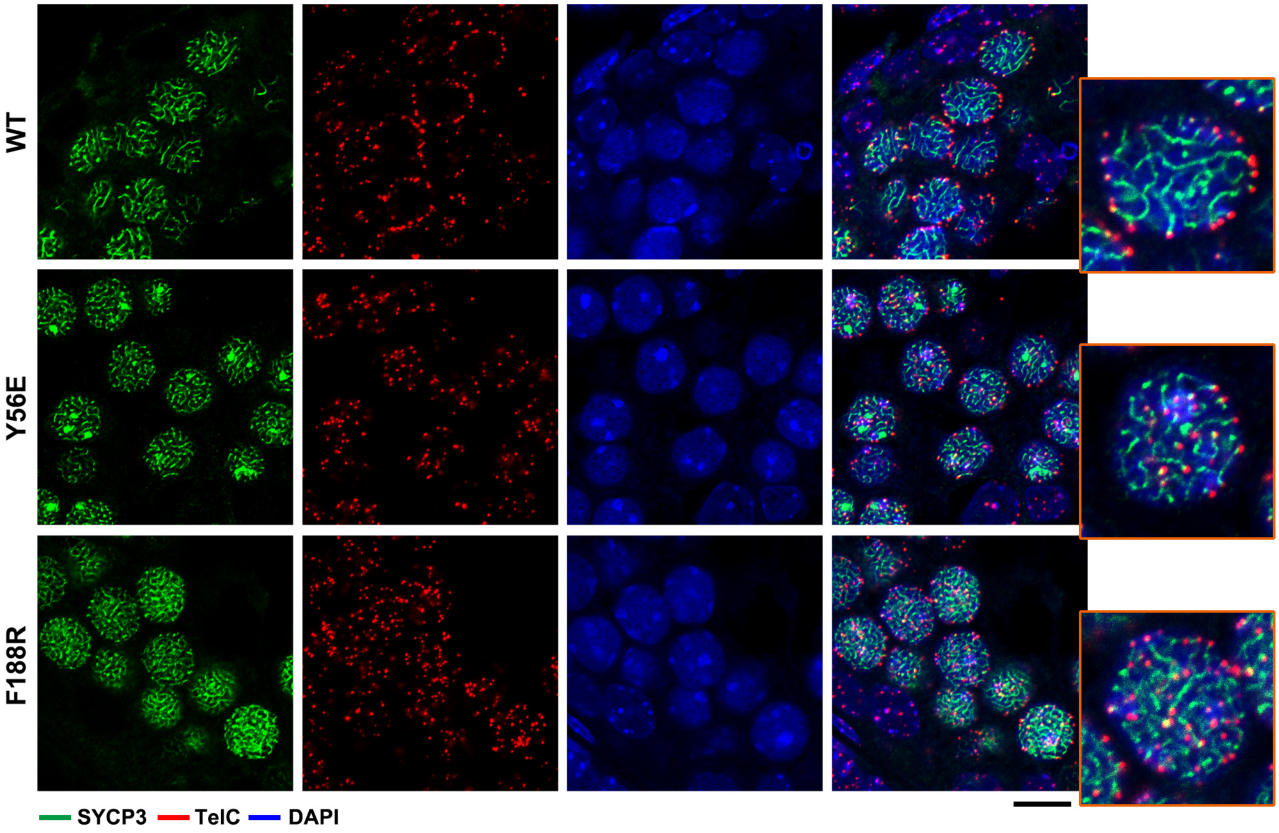
WT
Y56E
F188R
SYCP3
TelC
DAPI

Supplementary Figure 7

The telomeric-NE attachment is disrupted in TERB2-deficient spermatocytes

IF-FISH analysis of telomeres (TelC-FISH) and SYCP3 immunostaining on sections of two-week-old testes of different genotypes as indicated. The zoom-in views show a nucleus for each genotype. Scale bars, 10 μm.

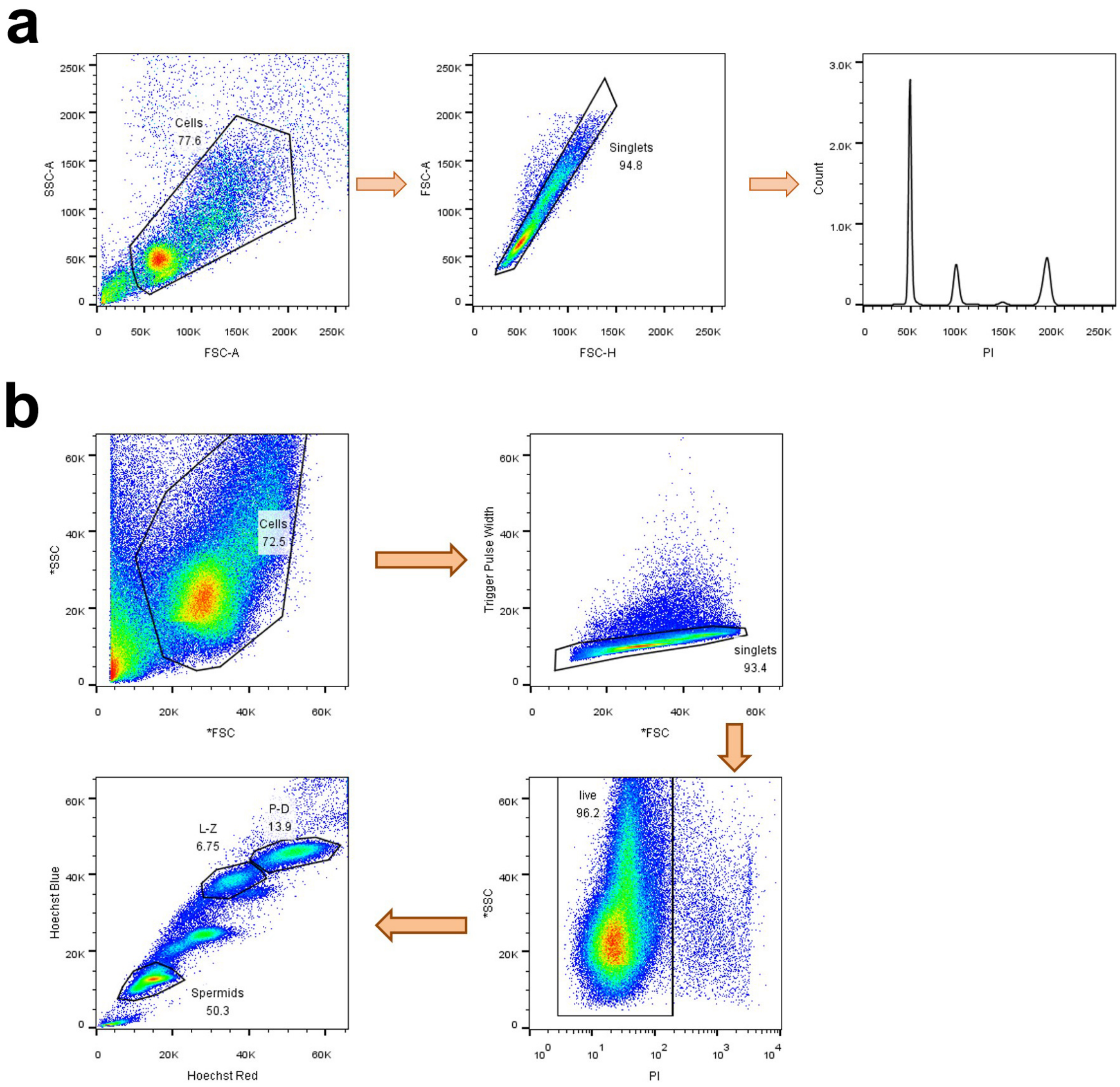
a
Cells
77.6
SSC-A
FSC-A
Singlets
94.8
FSC-A
FSC-H
Count
PI
b
Cells
72.5
*SSC
*FSC
Trigger Pulse Width
singlets
93.4
*FSC
live
96.2
*SSC
PI
L-Z
6.75
P-D
13.9
Spermids
50.3
Hoechst Blue
Hoechst Red

Supplementary Figure 8

FACS gating strategy examples

(a) Gating strategy example for Supplementary Figure 3b. **(b)** Gating strategy example for Supplementary Figure 3c.

Supplementary Table 1

Crystal data collection and refinement statistics

	$TERB2_{NTD}$-$TERB1_{TBM}$ (Native)	$TERB2_{NTD}$-$TERB1_{TBM}$ (Hg-SAD)
**Data collection**		
Wavelength	0.97853	0.97852
Space group	$P6_422$	$P6_422$
Cell dimensions		
a, b, c (Å)	126.9, 126.9, 90.4	124.8, 124.8, 89.3
α, β, γ (°)	90.0, 90.0, 120.0	90.0, 90.0, 120.0
Resolution (Å)	3.3	3.9
R_{merge}	0.076 (0.843) *	0.091 (1.188) *
I / σI	39.6 (2.4) *	264.0 (24.3) *
Completeness (%)	99.6 (96.1) *	100.0 (100.0) *
Redundancy	14.7 (9.9) *	246.2 (243.1) *
**Refinement**		
Resolution (Å)	46.96-3.30	
No. of reflections	5,905	
R_{work} / R_{free} (%)	28.9/33.7	
No. of atoms		
$TERB2_{NTD}$	1,669	
$TERB1_{TBM}$	993	
Ion	1	
B-factors		
$TERB2_{NTD}$	96.3	
$TERB1_{TBM}$	99.2	
Ion	126.8	
R.m.s. deviations		
Bond lengths (Å)	0.003	
Bond angles (°)	0.629	
Ramanchandran plot		
Favored region	91.4%	
Allowed region	100.0%	
Outlier region	0.0%	

*Highest resolution shell is shown in parenthesis

Supplementary Table 2

Crystal data collection and refinement statistics

	MAJIN_TERB2 (SeMet-SAD)
**Data collection**	
Wavelength	0.97776
Space group	*C*222
Cell dimensions	
a, b, c (Å)	107.7, 128.8, 86.9
α, β, γ (°)	90.0, 90.0, 90.0
Resolution (Å)	2.9
R_{merge}	0.077 (0.655) *
$I / \sigma I$	40.7 (3.4) *
Completeness (%)	99.9 (100.0) *
Redundancy	13.2 (13.5) *
**Refinement**	
Resolution (Å)	32.19-2.90
No. of reflections	13,729
R_{work} / R_{free} (%)	21.2/26.2
No. of atoms	
MAJIN	2,632
TERB2	800
B-factors	
MAJIN	88.2
TERB2	101.9
R.m.s. deviations	
Bond lengths (Å)	0.013
Bond angles (°)	1.751
Ramanchandran plot	
Favored region	93.4%
Allowed region	100.0%
Outlier region	0.0%

*Highest resolution shell is shown in parenthesis

Supplementary Table 3

Observed and expected frequencies of genotypes in mice born from heterozygous Y56E and F188R mice

	Y56E			F188R		
	**WT**	**Heterozygous**	**Homozygous**	**WT**	**Heterozygous**	**Homozygous**
**Observed no**	52 (24.9%)	99 (47.4%)	58 (27.7%)	43 (26.5%)	79 (48.8%)	40 (24.7%)
**Expected**	25%	50%	25%	25%	50%	25%
χ^2	0.92			0.21		

χ^2 is calculated by the sum of (observed-expected)^2/expected. The null hypothesis (H0) is that pups were born close to the expected Mendelian ratio. Degrees of freedom (df) are 2.

Supplementary Table 4

Fertility studies

Mutant males mated with WT females

**Mating**	**Y56E**		**F188R**	
	**heterozygous**	**homozygous**	**heterozygous**	**homozygous**
1	6	0	23	0
2	17	0	7	0
3	6	0	20	0
4	10	0	19	0
5	12	0	7	0
6	7	0	10	0

Mutant females mated with WT males

**Mating**	**Y56E**		**F188R**	
	**heterozygous**	**homozygous**	**heterozygous**	**homozygous**
1	6	0	5	0
2	7	0	10	0
3	6	0	8	0
4	4	0	2	0

For each genotype (Y56E heterozygous and homozygous, F188R heterozygous and homozygous) males (n=6) at 8 weeks were mated with two 8 week-old WT females for one consecutive month. After that females were sacrificed and the number of fetuses and pups was quantified. For each genotype (Y56E heterozygous and homozygous, F188R heterozygous and homozygous) females (n=4) at 8 weeks were mated with one 8 week-old WT females for one consecutive month. After that females were sacrificed and the number of fetuses and pups was quantified.
