# Supplementary material for: The meiotic TERB1-TERB2-MAJIN complex tethers telomeres to the nuclear envelope
Source: Nat Commun. 2019 Feb 4;10:564. doi: 10.1038/s41467-019-08437-1 (PMC6361898; doi:10.1038/s41467-019-08437-1)
Supplement: Supplementary file 4 — Reporting Summary [file 41467_2019_8437_MOESM4_ESM.pdf]

nature research

Corresponding author(s): Ming Lei

Reporting Summary

Nature Research wishes to improve the reproducibility of the work that we publish. This form provides structure for consistency and transparency in reporting. For further information on Nature Research policies, see Authors & Referees and the Editorial Policy Checklist.

Statistical parameters

When statistical analyses are reported, confirm that the following items are present in the relevant location (e.g. figure legend, table legend, main text, or Methods section).

n/a	Confirmed	
☐	☒	The exact sample size (n) for each experimental group/condition, given as a discrete number and unit of measurement
☐	☒	An indication of whether measurements were taken from distinct samples or whether the same sample was measured repeatedly
☐	☒	The statistical test(s) used AND whether they are one- or two-sided<br>*Only common tests should be described solely by name; describe more complex techniques in the Methods section.*
☒	☐	A description of all covariates tested
☒	☐	A description of any assumptions or corrections, such as tests of normality and adjustment for multiple comparisons
☐	☒	A full description of the statistics including central tendency (e.g. means) or other basic estimates (e.g. regression coefficient) AND variation (e.g. standard deviation) or associated estimates of uncertainty (e.g. confidence intervals)
☐	☒	For null hypothesis testing, the test statistic (e.g. F, t, r) with confidence intervals, effect sizes, degrees of freedom and P value noted<br>*Give P values as exact values whenever suitable.*
☒	☐	For Bayesian analysis, information on the choice of priors and Markov chain Monte Carlo settings
☒	☐	For hierarchical and complex designs, identification of the appropriate level for tests and full reporting of outcomes
☒	☐	Estimates of effect sizes (e.g. Cohen's d, Pearson's r), indicating how they were calculated
☐	☒	Clearly defined error bars<br>*State explicitly what error bars represent (e.g. SD, SE, CI)*

Our web collection on statistics for biologists may be useful.

Software and code

Policy information about availability of computer code

Data collection	N/A
Data analysis	FlowJoX (FlowJo, LLC), Imaris 9.0 (Bitplane)

For manuscripts utilizing custom algorithms or software that are central to the research but not yet described in published literature, software must be made available to editors/reviewers upon request. We strongly encourage code deposition in a community repository (e.g. GitHub). See the Nature Research guidelines for submitting code & software for further information.

Data

Policy information about availability of data

All manuscripts must include a data availability statement. This statement should provide the following information, where applicable:

- Accession codes, unique identifiers, or web links for publicly available datasets
- A list of figures that have associated raw data
- A description of any restrictions on data availability

The atomic coordinates and structure factors for the TERB1-TERB2 complex and the TERB2-MAJIN complex have been deposited to the Protein Data Bank (PDB) under the accession code PDB 6J07 and 6J08. The source data underlying Figs 4b, 4d, 4f, 4g, 5b, 5f, 5h, 6b, 6d, 7b, 7d, 7f, 7h, 8b, 8d, 8f, 9b, 9d, 10b, 10d and

Supplementary Figs 2e, 2f, 2g, 4b, 4d, 4f, 5b, 5e, 6b, 6d are provided as a Source Data file. Other data are available from the corresponding author upon reasonable request.

Field-specific reporting

Please select the best fit for your research. If you are not sure, read the appropriate sections before making your selection.

☒ Life sciences ☐ Behavioural & social sciences ☐ Ecological, evolutionary & environmental sciences

For a reference copy of the document with all sections, see nature.com/authors/policies/ReportingSummary-flat.pdf

Life sciences study design

All studies must disclose on these points even when the disclosure is negative.

Sample size	For animal experiments, no statistical method was used to predetermine sample size. For in vivo analysis, several cells (nuclei, spreads)or hundreds of telomeres were analyzed. This was shown to be sufficient in previous studies (e.g.,Shibuya et al Cell 2015, Mikolcevic et al Nat Commun 2016) to discern statistically significant differences.
Data exclusions	No data were excluded.
Replication	All attempts at replication were successful
Randomization	Animal experiments were not randomized. Mouse littermates were allocated into experimental groups by the genotypes.
Blinding	The investigators were blinded to the group allocation during experiments and outcome assessments.

Reporting for specific materials, systems and methods

Materials & experimental systems

n/a	Involved in the study	
☒	☐	Unique biological materials
☐	☒	Antibodies
☐	☒	Eukaryotic cell lines
☒	☐	Palaeontology
☐	☒	Animals and other organisms
☒	☐	Human research participants

Methods

n/a	Involved in the study	
☒	☐	ChIP-seq
☐	☒	Flow cytometry
☒	☐	MRI-based neuroimaging

Antibodies

Antibodies used	The following antibodies were used: rabbit polyclonal antibody against TERB1 (Long et al NSMB 2017, 1:1000), TERB2 (this study, 1:2000), MAJIN (this study, 1:2000), SUN1 (this study, 1:1000) , Speedy A (this study, 1:3000), phos-HistoneH2A.X (Ser139) (Millipore, 05-636-25UG, 1:2000), SYCP1 (Abcam, ab15087, 1:1000), SYCP3 (Abcam, ab15093, 1:2000), SOX9 (Millipore, ab5535, 1:200), MSH4 (Abcam, ab58666, 1:500), c-Myc (Santa Cruz, sc-789, 1:1000), TRF1 (Chen et al Science 2008, 1:1000), mouse monoclonal antibodies against MLH1 (BD, 550838, 1:200), SYCP3 (Abcam, ab97672, 1:2000), TRF1 (Abcam, ab66223, 1:1000), c-myc (Santa cruz, sc-40, 1:1000), FLAG (Sigma, F3165, 1:200), actin (Sigma, A2228, 1:2000), DMC1 (Abcam, ab11054, 1:100), Lamin B1 (Proteintech, 66095-1-Ig, 1:500), CDK2 (Santa curz, sc-6248, 1:100). Secondary antibody for western blot: Goat anti-Mouse IgG/HRP (Abclonal, AS003, 1:2000), Goat anti-Rabbit IgG/HRP (Proteintech, SA00001, 1:2000). Goat anti-mouse/rabbit secondary antibody for immunofluorescence: DyLight 488 (Thermo, 35502, 35553, 1:500), DyLight 550 (Thermo, 84540, 84541, 1:500), DyLight 633 (thermos, 35512, 35562, 1:500).
Validation	phos-HistoneH2A.X (Ser139) (Millipore, 05-636-25UG) Species Reactivity:Vertebrates,Applications: IHC IF WB ChIP<br>SYCP1 (Abcam, ab15087)Species Reactivity:Mouse Rat Human,Applications: IHC IF<br>SYCP3 (Abcam, ab15093)Species Reactivity:Mouse Rat Human,Applications: IHC IF<br>SOX9 (Millipore, ab5535)Species Reactivity:Mouse Rat Human,Applications: IHC IF WB<br>MSH4 (Abcam, ab58666)Species Reactivity:Mouse,Applications: ICC IF WB<br>c-Myc (Santa Cruz, sc-789)Species Reactivity:c-Myc tagged fusion proteins,Applications: WB, IP, IF<br>MLH1 (BD, 550838)Species Reactivity:Mouse Rat Human,Applications: IHC IF<br>SYCP3 (Abcam, ab97672)Species Reactivity:Mouse Hamster Zebrafish,Applications: IHC IF WB<br>c-myc (Santa cruz, sc-40)Species Reactivity:c-Myc tagged fusion proteins,Applications: WB, IP, IF<br>FLAG (Sigma, F3165)Species Reactivity:FLAG tagged fusion proteins,Applications: WB, IP, IF

actin (Sigma, A2228)Species Reactivity:Mouse Rat Human, Applications: WB, IHC
DMC1 (Abcam, ab11054)Species Reactivity:Mouse Rat Human, Applications: IHC IF WB
Lamin B1 (Proteintech, 66095-1-Ig)Species Reactivity:Mouse Rat Human, Applications: IHC IF WB
CDK2 (Santa curz, CDK2 (Santa curz, sc-6248)Species Reactivity:Mouse Rat Human, Applications: IF IP WB

Eukaryotic cell lines

Policy information about cell lines

Cell line source(s)	HEK293T and U-2 OS cells were obtained from the Cell Bank of Type Culture Collection of Chinese Academy of Sciences.
Authentication	Cells were authenticated by the cell bank of type culture collection of Chinese Academy of Sciences
Mycoplasma contamination	No mycoplasma contamination was detected in HEK293T and U-2 OS cells used in this study
Commonly misidentified lines (See ICLAC register)	No commonly misidentified cell lines were used

Animals and other organisms

Policy information about studies involving animals; ARRIVE guidelines recommended for reporting animal research

Laboratory animals	Fertilized eggs collected from C57BL/6 mice were used for generation of Terb2 mutant mice and Sun-/-mice by CRISPR/Cas-mediated genome engineering. Mutant mice were congenic with the C57BL/6 background. Testes and ovaries from mice at different ages as indicated in the manuscript were used for this study.
Wild animals	N/A
Field-collected samples	N/A

Flow Cytometry

Plots

Confirm that:

☒ The axis labels state the marker and fluorochrome used (e.g. CD4-FITC).

☒ The axis scales are clearly visible. Include numbers along axes only for bottom left plot of group (a 'group' is an analysis of identical markers).

☒ All plots are contour plots with outliers or pseudocolor plots.

☒ A numerical value for number of cells or percentage (with statistics) is provided.

Methodology

Sample preparation	Ecapsulated testes were dissociated by treatment of Collagenase type I followed by treatment of trypsin. Single-cell suspensions were then stained by Hoechst 33342 and PI. For PI staining of fixed cells, single-cell suspensions were fixed with ethanol prior to staining of PI.
Instrument	BD Influx cell sorter, BD FACSCalibur Flow Cytometer
Software	BD FACS sortware
Cell population abundance	The whole decapsulated testes were used to prepare single-cell suspensions
Gating strategy	Testicular cells were first gated by FSC-A vs SSC-A to eliminate any debris, then gated for singlets by FSC-H vs FSC-A or Trigger pulse width vs FSC-A. For live cell analysis, cells were further gated by PI staining to eliminate dead cells and spermatocytes were then determined from this gated population by Hoechst red vs Hoechst blue.

☒ Tick this box to confirm that a figure exemplifying the gating strategy is provided in the Supplementary Information.
